# Impaired HDL cholesterol efflux capacity in patients with non-alcoholic fatty liver disease is associated with subclinical atherosclerosis

**DOI:** 10.1038/s41598-018-29639-5

**Published:** 2018-08-03

**Authors:** Reza Fadaei, Hossein Poustchi, Reza Meshkani, Nariman Moradi, Taghi Golmohammadi, Shahin Merat

**Affiliations:** 1 Department of Clinical Biochemistry, Faculty of Medicine, Tehran University of Medical Sciences, Tehran, Iran; 2 Liver and Pancreatobiliary Diseases Research Center, Digestive Diseases Research Institute, Tehran University of Medical Sciences, Tehran, Iran; 3 Digestive Disease Research Center, Digestive Disease Research Institute, Tehran University of Medical Sciences, Tehran, Iran; 4 Department of Clinical Biochemistry, Faculty of Medicine, Kurdistan University of Medical Sciences, Sanandaj, Iran; 5 Department of Clinical Biochemistry, Faculty of Medicine, Iran University of Medical Sciences, Tehran, Iran

## Abstract

Non-alcoholic fatty liver disease (NAFLD) is associated with a substantial increased risk of atherosclerotic cardiovascular disease (ASCVD), which is partly related to dyslipidemia and low HDL-C level. The cardioprotective activity of HDL in the body is closely connected to its role in promoting cholesterol efflux, which is determined by cholesterol efflux capacity (CEC). Hitherto, the role of HDL, as defined by CEC has not been assessed in NAFLD patients. In this research study, we present the results of a study of cAMP-treated J774 CEC and THP-1 macrophage CEC in ApoB-depleted plasma of 55 newly diagnosed NAFLD patients and 30 controls. Circulating levels of ApoA-I, ApoB, preβ-HDL, plasma activity of CETP, PLTP, LCAT and carotid intima-media thickness (cIMT) were estimated. cAMP-treated J774 and THP-1 macrophage CEC were found to be significantly lower in NAFLD patients compared to controls (P < 0.001 and P = 0.003, respectively). In addition, it was discovered that both ApoA-I and preβ1-HDL were significantly lower in NAFLD patients (P < 0.001). Furthermore, cAMP-treated J774 CEC showed independent negative correlation with cIMT, as well as the presence of atherosclerotic plaque in NAFLD patients. In conclusion, our findings showed that HDL CEC was suppressed in NAFLD patients, and impaired cAMP-treated J774 CEC was an independent risk factor for subclinical atherosclerosis in NAFLD patients, suggesting that impaired HDL functions as an independent risk factor for atherosclerosis in NAFLD.

## Introduction

Non-alcoholic fatty liver disease (NAFLD) has emerged as the most common chronic liver disease worldwide, and includes a spectrum of liver disease conditions such as benign steatosis, inflammatory non-alcoholic steatohepatitis, fibrotic and cirrhotic liver disease conditions^1^. Numerous studies have revealed that NAFLD is associated with increased risk of atherosclerosis cardiovascular disease (ASCVD)^2–4^, which is the leading cause of death in NAFLD patients worldwide^5,6^. Several mechanisms have been proposed to elucidate the acceleration of atherosclerosis in NAFLD including insulin resistance, chronic inflammation, dysregulation of lipoprotein metabolism and atherogenic dyslipidemia^5,7^. Low high-density lipoprotein cholesterol (HDL-C) level, which is associated with atherogenic dyslipidemia in NAFLD, is a known risk factor for ASCVD^8,9^. HDL has several atheroprotective actions, such as enhancement of cholesterol efflux from peripheral tissues, anti-oxidant activity and anti-inflammatory activity^10^. However, recent studies have shown that HDL-C levels do not adequately predict ASCVD, and suggested that HDL function, particularly in cholesterol efflux capacity (CEC) might better explain the risk associated with low HDL^11,12^. Several cross-sectional studies to buttress this concept revealed that CEC is impaired in patients with coronary artery disease. Longitudinal studies conducted confirmed the relationship of decreased CEC with ASCVD, independent of HDL-C levels^11,13,14^. Several studies have shown atherogenic alterations in lipoprotein subclasses in relation to NAFLD^15–17^; however, until now there were no data on the relationship between CEC and subclinical atherosclerosis in NAFLD. Consequently, the aim of this study was to evaluate the relationship between CEC and subclinical atherosclerosis in NAFLD patients and control subjects.

## Results

### Anthropometric and metabolic profiles of the study population

Fifty-five patients with NAFLD and 30 control subjects participated in the present study. The participants were all males and matched in terms of age (P = 0.384). Body mass index (BMI) was higher in the NAFLD group compared to controls (P < 0.001). In addition, the number of overweight subjects (25 < BMI < 30) in patients with NAFLD (n [%] = 33 [60]) were higher than the controls (n [%] = 11 [36.7]) (P < 0.05). Similarly, the number of obese subjects (BMI > 30) in NAFLD patients (n [%] = 16 [29.1]) were higher compared to controls (n [%] = 3 [10]) (P < 0.05). Diastolic blood pressure (DPB) was higher in NAFLD group (P = 0.034). The systolic blood pressure (SBP) was higher in comparison with the control group but did not reach our threshold of statistically significant difference. NAFLD patients showed higher levels of insulin and homeostatic model assessment of insulin resistance (HOMA-IR) (P < 0.001 for both) compared to controls. Moreover, there were 7 patients with HOMA-IR > 2.4 in the NAFLD group while no subject had HOMA-IR > 2.4 in controls (P < 0.05). There was no significant difference in fasting blood glucose (FBG) between both groups. Patients with NAFLD demonstrated higher levels of triglycerides (TG) (P = 0.015) and lower levels of HDL-C (P = 0.025), and there was no significant difference in total cholesterol (TC) and low-density lipoprotein cholesterol (LDL-C) between both groups. Furthermore, 24 (43.6%) patients in NAFLD group and 8 (26.7%) control subjects showed TG > 150 mg/dL (P = 0.108). In addition, 2 (6.7%) control subjects and 10 (18.2%) NAFLD patients had HDL < 40 mg/dL (P = 0.145). As expected, liver function related tests showed higher levels of aspartate aminotransferase (AST) (P = 0.003), alanine aminotransferase (ALT) (P < 0.001) and gamma-glutamyltransferase (γ-GT) (P < 0.001) in NAFLD group compared to controls; however, alkaline phosphatase (ALP) showed no significant difference. In addition, liver stiffness (LS) as a measurement of liver fibrosis was higher in NAFLD patients compared to controls (P < 0.001). Furthermore, carotid intima-media thickness (cIMT), as a measurement of subclinical atherosclerosis was significantly increased in NAFLD patients (0.831 ± 0.11 mm) compared to controls (0.776 ± 0.09 mm) (P = 0.022). Details of the anthropometric and metabolic profiles of the study population are presented in Table 1.

**Table 1:** Anthropometric and metabolic profiles of study population.

Variable	Controls (n = 30)	NAFLD (n = 55)	P value
Age (year)	53.33 ± 8.12	51.84 ± 6.20	0.384
BMI (kg/m^2^)	24.60 ± 3.38	28.79 ± 3.74	<0.001
SBP (mmHg)	122 (110–138)	130 (119–141)	0.059
DBP (mmHg)	77.37 ± 10.93	82.91 ± 11.57	0.034
FBG (mg/dL)	89.81 ± 8.53	91.10 ± 5.95	0.466
Insulin (μU/mL)	3.28 ± 1.98	6.74 ± 3.09	<0.001
HOMA-IR	0.73 ± 0.45	1.52 ± 0.71	<0.001
TG (mg/dL)	124.5 ± 39.20	152.4 ± 63.86	0.015
TC (mg/dL)	184.9 ± 30.61	197.5 ± 38.05	0.123
LDL-C (mg/dL)	108.1 ± 27.12	115.2 ± 27.86	0.260
HDL-C (mg/dL)	50.85 ± 9.03	46.57 ± 7.79	0.025
AST (U/L)	18 (15.2–22)	22.9 (18–29.1)	0.003
ALT (U/L)	16.2 (13–21.4)	26.7 (18–39.6)	<0.001
ALP (U/L)	227.3 ± 58.49	228.1 ± 61.17	0.957
ɣ-GT (U/L)	19.7 (15.8–25.8)	29.2 (22–36.6)	<0.001
Urea nitrogen (mg/dL)	29.46 ± 6.59	30.34 ± 7.52	0.594
Creatinine (mg/dL)	1.15 ± 0.10	1.19 ± 0.11	0.099
Liver Stiffness (kPa)	3.6 (3–4.3)	5.1 (4–6.1)	<0.001
cIMT (mm)	0.776 ± 0.09	0.831 ± 0.11	0.022

Data are shown in mean ± SD for normal distributed variables and median (inter quartile range) for non-normal distributed variables. BMI, Body Mass Index; SBP, Systolic Blood Pressure; DBP, Diastolic Blood Pressure; FBG, Fasting Blood Glucose; TG, Triglyceride; TC, Total Cholesterol; LDL-C, Low Density Lipoprotein-Cholesterol; HDL-C, High Density Lipoprotein; AST, Aspartate amino Transferase, ALT, Alanine amino Transferase; ALP, Alkaline Phosphatase; ɣ-GT, Gamma-Glutamyl transferase; cIMT; carotid Intima Media Thickness.

### Apolipoprotein levels, enzyme activities and CECs

**Figure 1:**
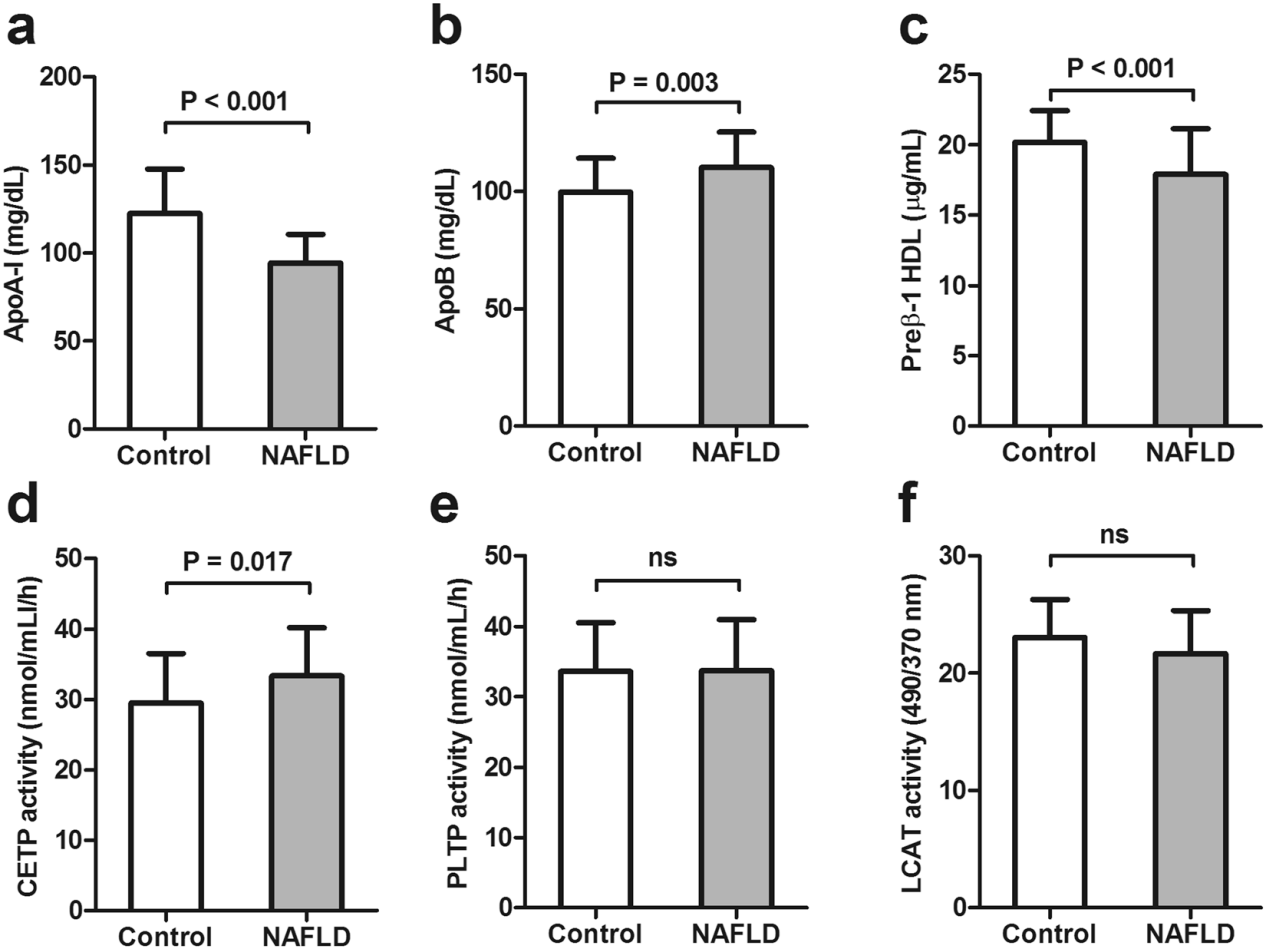
Apolipoproteins levels, enzyme activities. (**a**) Apolipoprotein A-I (ApoA-I) plasma concentration in NAFLD group (94.5 ± 2.2 [mg/dL]) was lower than controls (122.6 ± 4.6 [mg/dL]). (**b**) Plasma apolipoprotein B (Apo B) in NAFLD group (110.18 ± 2.04 [ng/mL]) demonstrated higher concentration compared with controls (99.75 ± 2.63 [mg/dL]). (**c**) Prebeta1-HDL (Preβ1-HDL) level in NAFLD (17.89 ± 0.44 [µg/mL]) showed significant decrease compared with controls (20.17 ± 0.41 [µg/mL]). (**d**) Activity of Cholesterol Ester Transferase Protein (CETP) in NAFLD (33.34 ± 0.93 [nmol/mL/h]) demonstrated significant increase compared with controls (29.50 ± 1.29 [nmol/mL/h]). (**e**) Activity of Plasma Lipid Transfer Protein (PLTP) in controls was an average of 33.55 ± 1.26 nmol/mL/h, in NAFLD was 33.71 ± 0.98 nmol/mL/h that showed no significant difference. (**f**) Activity of Lecithin Cholesterol Acyl Transferase (LCAT) showed no significant difference between controls (23.07 ± 0.59 [490/370 nm]) and NAFLD group (21.69 ± 0.49 [490/370 nm]). Data are given with mean ± SD.

**Figure 2:**
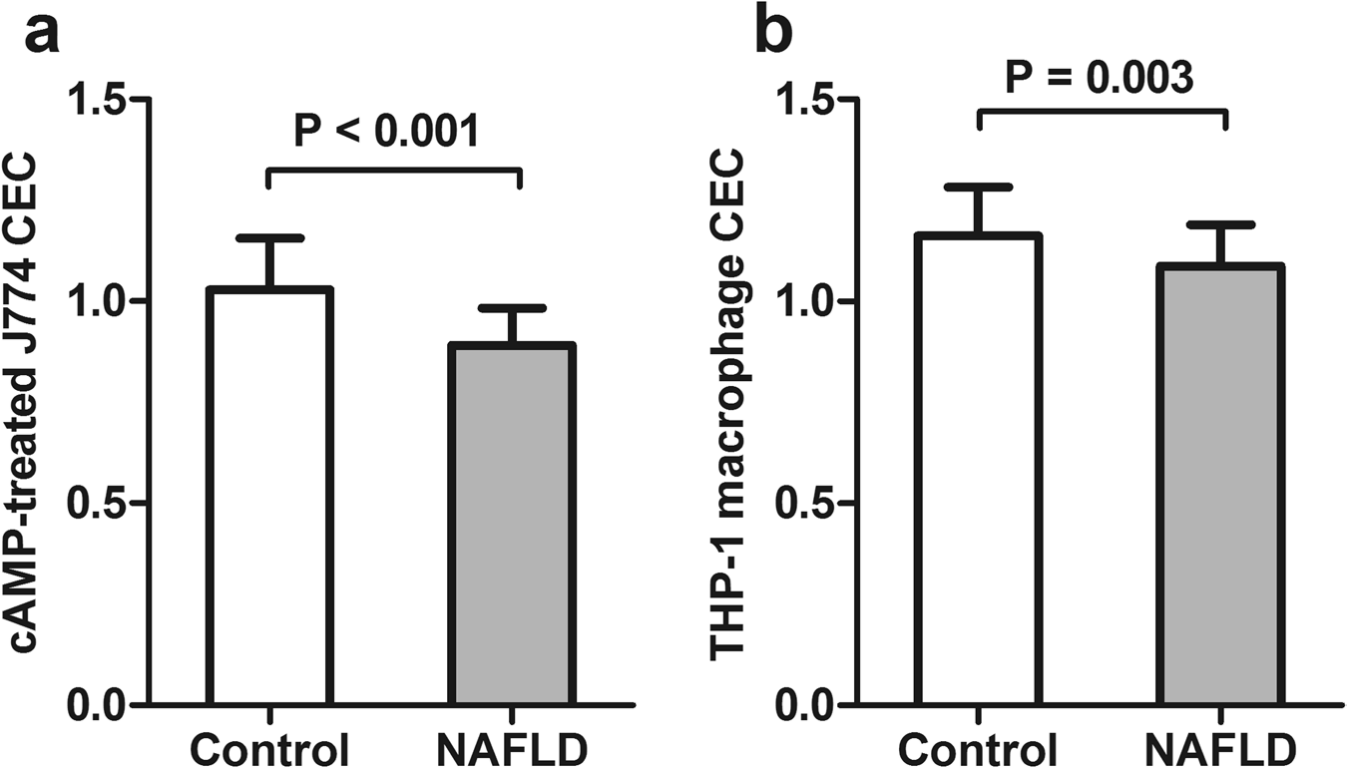
cAMP-treated J774 and THP-1 macrophage Cholesterol Efflux Capacity (CEC). (**a**) cAMP-treated J774 CEC showed significant decrease in NAFLD group compared with controls, before and after adjustment for age, BMI, HOMA-IR, TG, HDL-C (P < 0.001 and P = 0.001, respectively). (**b**) THP-1 macrophage CEC demonstrated significant decrease in NAFDL compared with controls (P = 0.003), but after adjustment for age, BMI, HOMA-IR, TG, HDL-C the significant difference was disappeared (P = 0.253). Data are shown in mean ± SD.

Circulating apolipoprotein A-I (ApoA-I) levels were lower in NAFLD patients (94.5 ± 16.16 vs 122.6 ± 25.2 mg/dL, P < 0.001) compared to controls (Fig. 1a), while apolipoprotein B (ApoB) levels was found to be higher in NAFLD patients (110.18 ± 15.14 vs 99.75 ± 14.41 mg/dL, P = 0.003) compared to controls (Fig. 1b). In addition, it was found that preβ1-HDL decreased in NAFLD group (17.89 ± 3.27 vs 20.17 ± 2.25 µg/mL, P < 0.001) compared to controls (Fig. 1c). Our results showed that plasma activity of cholesterylester transfer protein (CETP) increased in NAFLD patients (33.34 ± 6.86 nmol/mL/h) compared to controls (29.50 ± 7.05 nmol/mL/h, P = 0.017) (Fig. 1d), but there was no significant difference in the activities of phospholipid transfer protein (PLTP) and lecithin–cholesterol acyltransferase (LCAT) between the two groups (Fig. 1e,f). In addition, cAMP-treated J774 CEC decreased significantly in NAFLD patients (0.89 ± 0.09) compared to controls (1.03 ± 0.13) (P < 0.001). Furthermore, our results demonstrated a significant decrease in THP-1 macrophage CEC in patients with NAFLD (1.09 ± 0.10) compared to controls (1.16 ± 0.12) (P = 0.003) (Fig. 2b). Moreover, analysis of covariance was performed to adjust the effect of HDL-C and ApoA-I on HDL CECs, and the results showed that decrease in cAMP-treated J774 CEC was independent of HDL-C and ApoA-I levels (P = 0.029). However, the significant difference shown by THP-1 macrophage CEC disappeared after adjustment of HDL-C and ApoA-I levels (P = 0.129). Additionally, taking into account the increase in BMI, insulin resistance, TG and TC and lower levels of HDL-C in NAFLD patients, we performed additional analyses of covariance in order to adjust the effect of these variables. Our data indicated that the differences in cAMP-treated J774 CEC were independent of BMI, HOMA-IR, TC, TG and HDL-C (P = 0.001), but the difference between the two groups for THP-1 macrophage CEC was no longer statistically significant after adjustment (P = 0.232).

### Relationship of CECs with anthropometric and biochemical parameters

Correlation analysis was performed for both HDL CECs, and the results are presented in Table 2. cAMP-treated J774 CEC showed positive correlation with THP-1 macrophage CEC in all participants; both HDL CECs had positive correlations with HDL-C, ApoA-I, preβ1-HDL and LCAT activity. Conversely, cAMP-treated J774 CEC showed significant inverse correlations with BMI, insulin, HOMA-IR and LS. Multivariate stepwise linear regression analysis carried out with cAMP-treated J774 CEC as dependent variable indicated that ApoA-I (β = 0.590, P < 0.001) and HDL-C (β = 0.236, P = 0.006) are two independent determinants of cAMP-treated J774 CEC in all participants. When ApoA-I was excluded from the model, preβ1-HDL (β = 0.384, P < 0.001) and HDL-C (β = 0.335, P = 0.001) were still observed as two independent factors associated with cAMP-treated J774 CEC. Also, HDL-C (β = 0.462, P < 0.001) and preβ1-HDL (β = 0.276, P = 0.004) were two independent predictors of THP-1 macrophage CEC in all participants. Moreover, correlation analysis in controls revealed that cAMP-treated J774 CEC correlated positively with HDL-C and ApoA-I, and THP-1 macrophage CEC showed a positive correlation with HDL-C. However, in NAFLD patients, THP-1 macrophage CEC demonstrated a positive correlation with cAMP-treated J774 CEC, and both CECs had a positive correlation with HDL-C, preβ-HDL and LCAT activity. In addition, cAMP-treated J774 CEC showed a positive correlation with ApoA-I and negative correlation with FBG and LS. Multivariate stepwise linear regression analysis with all correlated parameters showed that ApoA-I (β = 0.481, P < 0.001) and LCAT activity (β = 0.307, P = 0.008) level were two significant predictors of cAMP-treated J774 CEC. Also, HDL-C (β = 0.386, P = 0.002) and preβ-HDL (β = 0.289, P = 0.020) level were two significant predictors of THP-1 macrophage CEC in NAFLD patients. Age adjusted correlations for both HDL CECs are shown in Supplementary Table S1.

**Table 2:** Pearson correlation of cAMP-treated J774 CEC and THP-1 macrophage CEC with anthropometric and metabolic profiles.

Variables	cAMP-treated J774 CEC			THP-1 macrophage CEC		
	All Participant	Controls	NAFLD	All Participant	Controls	NAFLD
Age	0.126	0.199	−0.028	0.094	0.091	0.043
BMI	−0.303^**^	0.051	−0.136	−0.270^*^	−0.257	−0.072
FBG	−0.199	−0.085	−0.280^*^	−0.035	0.104	−0.105
Insulin	−0.272^*^	0.095	−0.037	−0.160	0.025	0.001
HOMA-IR	−0.286^**^	0.103	−0.077	−0.152	0.057	0.001
TG	−0.185	−0.201	−0.026	−0.129	−0.178	−0.017
TC	−0.181	−0.208	−0.056	0.036	−0.005	0.148
LDL-C	−0.100	−0.133	0.025	0.085	−0.046	0.241
HDL-C	0.441^**^	0.408^*^	0.358^**^	0.538^**^	0.568^**^	0.453^**^
SBP^a^	−0.094	−0.131	0.087	0.002	0.218	−0.035
DBP	−0.162	−0.062	−0.041	−0.092	−0.025	−0.016
Urea nitrogen	−0.057	−0.178	0.005	0.014	−0.011	0.059
Creatinine	0.032	0.083	0.171	−0.032	−0.023	0.064
AST^a^	−0.122	0.361	−0.085	−0.068	0.289	−0.064
ALT^a^	−0.187	0.266	−0.066	−0.167	0.201	−0.142
ALP	−0.006	−0.033	0.018	−0.033	−0.002	−0.051
ɣ-GT^a^	−0.173	0.217	−0.049	−0.111	0.085	−0.012
LS^a^	−0.323**	0.221	−0.312*	−0.087	−0.149	0.193
ApoB	−0.101	0.135	0.054	−0.088	−0.161	0.121
ApoA-I	0.672^**^	0.530^**^	0.534^**^	0.323^**^	0.239	0.129
preβ1-HDL	0.470^**^	0.243	0.460^**^	0.404^**^	0.241	0.377^**^
CETP activity	−0.111	0.011	0.049	−0.033	0.032	0.070
PLTP activity	0.044	0.039	0.075	−0.022	−0.005	−0.028
LCAT activity	0.344^**^	0.169	0.389^**^	0.334^**^	0.287	0.302^*^
cAMP-treated J774 CEC	—	—	—	0.412^**^	0.307	0.298^*^

*p < 0.05 and **p < 0.01.

^a^Logarithmic transformation was performed.

### Relationship of cIMT with anthropometric and biochemical parameters

The correlation analysis of cIMT with anthropometric and metabolic profile are given in Table 3. Correlation analysis in all participants showed that cIMT correlated positively with insulin, HOMA-IR, TC and LS, and correlated negatively with HDL-C, ApoA-I, pre-B-HDL, LCAT activity, cAMP-treated J774 CEC and THP-1 macrophage CEC. Multivariate linear regression analysis with cIMT as dependent variable showed no significant prediction of cIMT in all participants. Subgroup analysis in controls showed that cIMT had a positive correlation with age and SBP; in the multivariate linear regression analysis, SBP (β = 0.473, P = 0.011) was a significant predictor of cIMT. Furthermore, in NAFLD patients, cIMT showed a positive correlation with TC and a negative correlation with ApoA-I, preβ1-HDL and cAMP-treated J774 CEC. Strikingly, when the variables correlated with cIMT were added to the multivariate linear regression analysis, cAMP-treated J774 CEC (β = −0.395, P = 0.005) and TC (β = 0.257, P = 0.028) were two independent predictors of cIMT in NAFLD patients (Model 1, Table 4). Taking into account the correlation of cAMP-treated J774 CEC with LCAT activity, LS, FBG and HDL-C, additional adjusted linear regression analysis was conducted to remove the possible effect of these variables (Model 2, Table 4). Results showed that TC (β = 0.271, P = 0.029) and cAMP-treated J774 CEC (β = −0.385, P = 0.022) remained as two independent predictors of cIMT in NAFLD patients. In addition, we performed coronary artery disease risk factor adjusted linear regression analysis (Model 3, Table 4), and cAMP-treated J774 CEC (β = −0.328, P = 0.026) remained as the only significant predictor of cIMT in patients with NAFLD. Age adjusted correlations for cIMT are presented in Supplementary Table S2.

**Table 3:** Pearson correlation of cIMT with anthropometric and metabolic profiles.

Variable	All Participant	Controls	NAFLD
Age	0.182	0.363^*^	0.141
BMI	0.186	0.215	0.024
FBG	0.055	−0.146	0.147
Insulin	0.231^*^	−0.291	0.239
HOMA-IR	0.244^*^	−0.309	0.265
TG	0.212	−0.095	0.233
TC	0.262^*^	0.104	0.274^*^
LDL-C	0.209	0.121	0.214
HDL-C	−0.308^**^	−0.296	−0.254
SBP^a^	0.144	0.534^**^	−0.073
DBP	0.083	0.275	−0.073
Urea nitrogen	0.058	0.297	−0.050
Creatinine	0.065	−0.125	0.075
AST^a^	0.183	0.056	0.129
ALT^a^	0.083	−0.132	0.009
ALP	−0.031	0.249	−0.150
ɣ-GT^a^	−0.022	−0.065	−0.159
LS^a^	0.226*	0.033	0.164
ApoB	0.171	0.057	0.117
ApoA-I	−0.428^**^	−0.353	−0.397^**^
preβ1-HDL	−0.389^**^	−0.217	−0.369^**^
CETP	0.126	−0.036	0.112
PLTP	0.176	0.178	0.181
LCAT	−0.238^*^	−0.294	−0.166
cAMP-treated J774 CEC	−0.490^**^	−0.334	−0.522^**^
THP-1 macrophage CEC	−0.246^*^	−0.163	−0.194

*p < 0.05 and **p < 0.01.

^a^Logarithmic transformation was performed.

**Table 4:** Multivariate linear regression (MLR) with cIMT as independent predictor in NAFLD patients.

Variables	Model 1		Model 2		Model 3	
	β	p	Β	P	β	P
TC	0.257	0.028	0.271	0.029	0.168	0.543
ApoA-I	−0.086	0.585	−0.088	0.624	−0.110	0.535
preβ1-HDL	−0.146	0.332	−0.111	0.529	−0.170	0.275
cAMP-treated J774 CEC	−0.395	0.005	−0.385	0.022	−0.328	0.026

Model (1) MLR with all correlated parameters. Model (2) Adjusted MLR for LCAT activity, LS, FBG, THP-1 macrophage CEC and HDL-C. Model (3) Adjusted MLR for age, BMI, HOMA-IR, TG, LDL-C, HDL-C, SBP and DBP.

β: Standardized coefficient.

### Relationship of CECs with carotid atherosclerotic plaque

**Figure 3:**
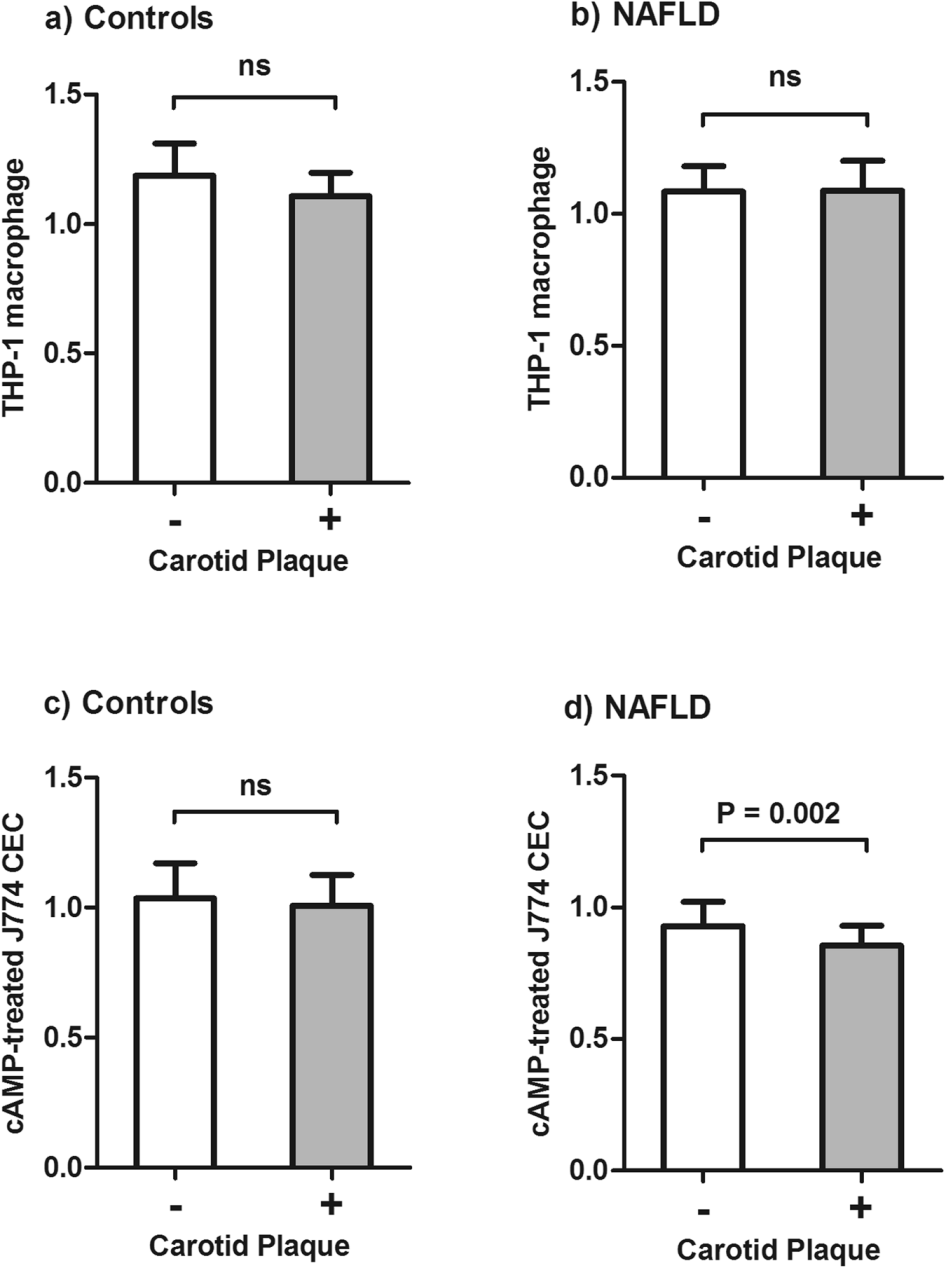
HDL CECs according to presence of atherosclerotic plaque. (**a**) THP-1 macrophage CEC in controls according to presence of atherosclerotic plaque. (**b**) THP-1 macrophage CEC in NAFLD patients according to presence of atherosclerotic plaque. (**c**) cAMP-treated J774 CEC in NAFLD patients with and without atherosclerotic plaque. (**d**) cAMP-treated J774 CEC in NAFLD according to presence of atherosclerotic plaque.

**Figure 4:**
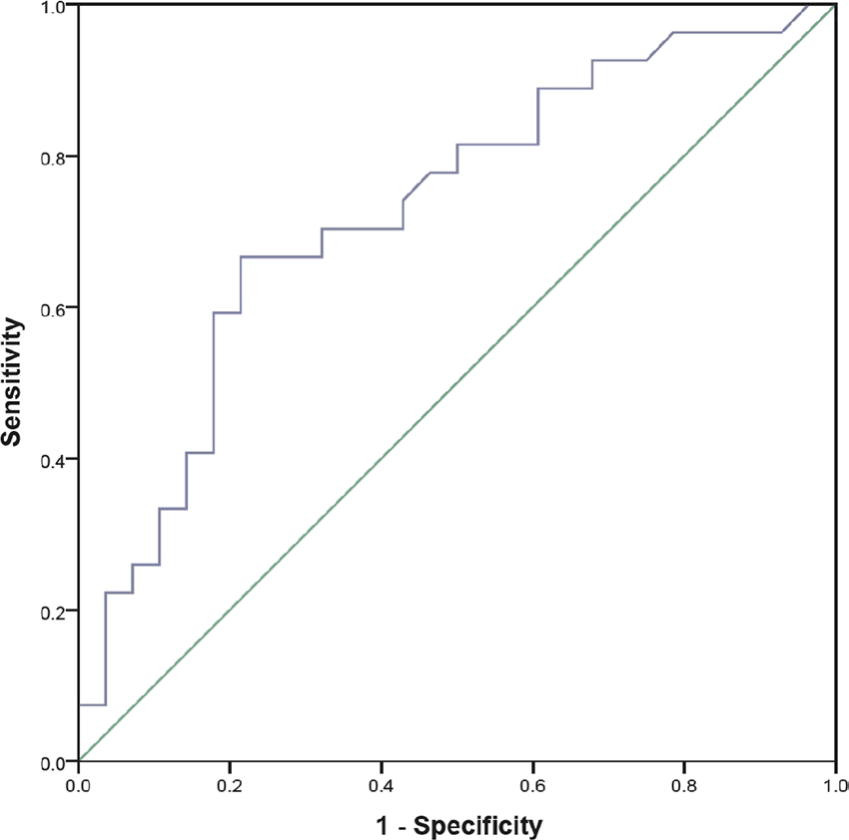
ROC curve for diagnostic of presence of atherosclerotic plaque in NAFLD according to cAMP-treated J774 CEC. The area under curve was 0.728 (95% CI = 0.593–0.863, P = 0.004).

We analyzed the association of CECs and carotid atherosclerotic plaque in the study population. As shown in Fig. 3, THP-1 macrophage CEC showed no significant difference in controls with atherosclerotic plaque (n = 9) (1.108 ± 0.088) compared to controls with no atherosclerotic plaque (n = 21) (1.187 ± 0.124, P = 0.097). In addition, THP-1 macrophage CEC in NAFLD patients demonstrated no significant difference between patients with (n = 28) (1.089 ± 0.111) and without atherosclerotic plaque (n = 27) (1.084 ± 0.097, P = 0.866). Furthermore, cAMP-treated J774 CEC in controls with atherosclerotic plaque (1.009 ± 0.117) showed no significant difference compared to controls without atherosclerotic plaque (1.037 ± 0.134, P = 0.591). However, cAMP-treated J774 CEC was significantly lower in NAFLD patients with atherosclerotic plaque (0.854 ± 0.076) compared to NAFLD patients without atherosclerotic plaque (0.929 ± 0.092, P < 0.002). In addition, logistic regression analysis was conducted to evaluate the risk of atherosclerotic plaque in NAFLD patients according to cAMP-treated J774 CEC. In crude logistic regression analysis, it was found that cAMP-treated J774 CEC was associated with atherosclerotic plaque (β = −9.88, P = 0.006). In addition, we adjusted the effect of LCAT activity, LS, FBG, THP-1 macrophage CEC and HDL-C as correlated factors with cAMP-treated J774 CEC. Additional adjustment for CAD risk factors was also performed. In both adjusted models, cAMP-treated J774 CEC was independently associated with the presence of atherosclerotic plaque in NAFLD patients (β = −11.62, P = 0.022 and β = −15.09, P = 0.003, respectively). Also, receiver operating characteristic (ROC) curve analysis was plotted to assess the diagnostic value of cAMP-treated J774 CEC as a biomarker of atherosclerotic plaque in NAFLD patients. The result showed that the area under the curve representing decrease in cAMP-treated J774 CEC was 0.728 (95% CI = 0.593–0.863, P = 0.004) (Fig. 4).

## Discussion

The major findings of the present study include decrease in cAMP-treated J774 CEC in NAFLD patients, negative independent association of cAMP-treated J774 CEC with cIMT and the presence of atherosclerotic plaque in NAFLD patients. To the best of our knowledge, the present study is the first to evaluate CEC and its relationship with subclinical atherosclerosis in patients with NAFLD.

Interestingly, some previous studies have found increased CEC in metabolic syndrome (MetS) and insulin-resistant subjects^18–20^. A study by Nestel *et al*., showed increased CEC in overweight MetS subjects with insulin resistance compared to overweight MetS subjects without insulin resistance^19^. In the above study, whole plasma was used as the acceptor, but in the present study, ApoB-depleted plasma was employed. In addition, the Nestel *et al*., study did not include a healthy control and the authors used a LXR agonist to upregulate cholesterol transporters, whereas in the present study we had healthy controls and used modified LDL to upregulate cholesterol transporters in THP-1 macrophage CEC. A separate study conducted with 15 controls and 35 MetS subjects showed increased CEC in MetS, but they used transfected Baby Hamster Kidney cells to express *ABCA1* and *ABCG1*^21^. In contrast, in the present study, we used two well established macrophage cell lines for CEC assessments. As regards CVD, it is preferable to use macrophage cell line for cholesterol efflux assay^11,13,14^. In addition, it was found that ApoB-depleted plasma for CEC maintained preβ1-HDL as an acceptor for ABCA1-mediated cholesterol efflux, which is highly relevant for the pathogenesis of atherosclerosis^22,23^. Overall, the differences in the study populations and methods could account for the differences between the present study and the cited studies.

However, in the context of MetS and diabetes, several studies reported a decrease in CECs using a wide range of acceptors and cellular systems^24–28^. A large cohort study with 1202 participants showed that CEC was correlated with the presence of MetS^29^. The findings of this study are consistent with a study that reported decreased CEC in patients with MetS; in that study, they used THP-1 cell line treated with modified LDL and ApoB-depleted plasma^30^.

Furthermore, in patients with cirrhosis, Trieb *et al*., demonstrated a significant change in HDL functions^31^; they specifically reported that ABCA1-mediated CEC was significantly suppressed in patients with cirrhosis, while CEC from isolated HDL, which is mainly ABCA1 independent showed no significant difference^31^. However, in Trieb *et al*., study, cirrhosis was a consequence of different factors, including viral infection and alcohol consumption. In above-mentioned study, patients with significant liver damage were included. In contrast, our study was conducted on patients with early NAFLD (not cirrhosis). Despite these differences, our results demonstrated independent decrease in cAMP-treated J774 CEC in NAFLD patients, which is consistent with the findings of Trieb *et al*., study. However, the significant decrease in THP-1 macrophage CEC in NAFLD patients was eliminated after adjustment for ApoA-I and HDL-C.

In addition, consistent with the Trieb *et al*., study, our results demonstrated a significant decrease in ApoA-I and preβ1-HDL in NAFLD patients. It is well known that lipid-poor ApoA-I and preβ1-HDL are the main acceptors for ABCA1-mediated CEC^23^. It has been shown that cAMP upregulates ABCA1 in J774 macrophage^32^. Our results showed that ApoA-I and preβ1-HDL were the two variables that had the strongest correlations with cAMP-treated J774 CEC, and that the correlation with THP-1 macrophage CEC was weaker compared to those with cAMP-treated J774 CEC. Moreover, subgroup analysis revealed that the correlations of cAMP-treated J774 with ApoA-I and preβ1-HDL in NAFLD patients were stronger than controls, and that ApoA-I and HDL-C were independents predictors for cAMP-treated J774 CEC in NAFLD patients. These results suggest that decreased ApoA-I and preβ1-HDL levels might elucidate the decrease in CECs, particularly in the case of cAMP-treated J774 CEC in NAFLD patients. Furthermore, HDL-C demonstrated significant correlations with both CECs in all subgroups, but the correlation with THP-1 macrophage CEC was stronger than that with cAMP-treated J774 CEC. Both HDL CECs showed positive correlations with LCAT activity; LCAT activity level showed an independent association with cAMP-treated J774 CEC in NAFLD patients. The precise role of LCAT activity in CEC^33,34^ is a subject of ongoing debate and study, but considering its role in esterifying cholesterol^35^, and the positive correlations of LCAT activity and CEC in our study population, our data suggest a positive role for LCAT in cholesterol efflux.

The impact of insulin resistance and obesity on CEC^18,20,21,25^ remain unclear, with one study asserting that insulin resistance^19^ promotes increased CEC, while another study suggested a role for hypertriglyceridemia in increased CEC^36^, and yet other studies reported negative or no correlation between features of MetS and CEC^24,29^. Our results showed inverse correlations between cAMP-treated J774 CEC and BMI, HOMA-IR and insulin in the whole population, and positive correlation with FBG in NAFLD patients. However, multivariate linear regression analysis showed no independent correlation of CEC with BMI, HOMA-IR or insulin level; HDL-C, as a component of MetS had significant correlation with CECs. Overall, our results showed that low HDL-C level was the most important component of MetS, having independent association with CECs. In addition, LS as a biomarker for liver fibrosis had negative correlation with cAMP-treated J774 CEC in all participants; in NAFLD patients, there was no independent correlation between cAMP-treated J774 CEC and LS. This result suggests a possible role for liver fibrosis in decreased CEC, but more studies are needed to substantiate this concept.

Another interesting finding of the present study was that cAMP-treated J774 CEC had an independent correlation with subclinical atherosclerosis in NAFLD patients. Our results showed that cAMP-treated J774 CEC and TC were two independent predictors of cIMT in NAFLD patients. In an adjusted model for CAD risk factor, cAMP-treated J774 CEC was the only variable with significant correlation with cIMT. Several studies have reported an independent association between biomarkers of atherosclerosis and coronary artery disease with CEC in cardiometabolic disease^11,13,14^. Most of these studies employed a similar system for CEC, and it has been suggested that cAMP-treated J774 CEC may have stronger relationship with atherosclerosis^11,13,23^. Strikingly, our findings for the first time showed that impaired cAMP-treated J774 CEC had an independent association with subclinical atherosclerosis in NAFLD patients. Importantly, our data were obtained from patients who were newly diagnosed with NAFLD and who were consequently free from potentially confounding effects of antihypertensives, lipid and/or glucose lowering medications. A limitation of the present study was that only men were included; therefore, our results should carefully be generalized considering the fact that women were not included.

In conclusion, our findings showed that the substrate for ABCA1-mediated CEC (i.e. ApoA-I and preβ1-HDL) was significantly suppressed in NAFLD patients indicating that ApoA-I and preβ1-HDL could be the cause of the impairment observed in HDL CECs. In addition, the impaired cAMP-treated J774 CEC is an independent risk factor for increased manifestation of subclinical atherosclerosis as defined by increased cIMT and presence of atherosclerotic plaque in NAFLD patients.

## Subjects and Methods

### Study population

This case-control study was conducted on 55 newly diagnosed NAFLD patients, recruited from the outpatient clinic of Shariati Hospital (Tehran, Iran), and 30 healthy volunteers with no metabolic disorder, as well as normal levels of liver function tests and ultrasonography assessment, which were included as controls. All participants were aged between 42 and 72 years. NAFLD was diagnosed employing ultrasonography and fibroscan, a non-invasive assessment of liver fibrosis, performed on all participants. All subjects were free of any medications causing steatosis, modifying lipids, lowering glucose and antihypertensive. Also, exclusions criteria included having diabetes mellitus, infectious disease, malignancy and any other liver disease and a history of alcohol consumption more than 30 g/day. Diabetes mellitus was diagnosed according to the criteria of the American Diabetes Association^37^. All participants provided informed written consent, and this study was conducted in accordance with the “Declaration of Helsinki” and approved by the Ethics Committee of Tehran University of Medical Sciences.

### Ultrasonography and elastography

Ultrasonography was performed to assess the carotid arteries and liver by Accuvix XQ (Madison, South Korea), equipped with a 3–7 MHz curved transducer and a 5–12 MHz linear array transducer, as previously described^38^. Ultrasonography scoring system was employed to determine fatty liver, since it provides high specificity (100%) and sensitivity (91.7%) in the diagnosis of fatty liver^39^. In this protocol, ultrasonography results included vascular blurring (score 0 to 1), hepatorenal echo contrast and/or liver brightness (score 0 to 3) and deep attenuation (score 0 to 2). A total score of at least 2 was required for diagnosis of NAFLD. In addition, controls had a score less than 1. Furthermore, cIMT was computed as the average of right and left cIMT. LS was measured by transient elastography employing FibroScan® 502 machine (EchoSense, France, 5 MHz) and X and XL probes, as previously described^38^.

### Anthropometric and laboratory measurement

Study participants were aged between 42 and 72 years. Height and weight were measured for calculation of BMI using the following equation: body weight (kg)/square of height (m^2^). Fasting blood samples were obtained after an overnight fasting. FBG, LDL, HDL, TC, TG, urea, Cr, ALT, AST, ALP and ɣ-GT were measured employing commercial kits (Pars azmoon, Iran) and by an auto analyzer. ELISA kit (Monobind, USA) was used to measure fasting insulin. HOMA-IR was computed using the following equation: [FBG (mg/dL)] × [fasting blood insulin (μU/mL)/405].

### Determination of cholesterol efflux

ApoB-depleted plasma was provided using polyethylene glycol (6000) (Sigma, USA) in 10 mM HEPES (pH = 8) after centrifugation at 2200 g in 4 °C for 30 min, as previously described^11,13,14^. The supernatant containing HDL was used for Cholesterol Efflux Assay (CEA). CEC was measured using two validated cellular systems, (i) cAMP-treated J774 murine macrophages and (ii) THP-1 macrophages CEC, as previously described^11,14,30^. J774 murine macrophage were plated and radiolabeled with 1 µCi ^3^H-Cholesterol (Perkin Elmer, USA). The cells were incubated with 8-(-4-chlorophenylthio)-cAMP for 6 hours to induce of *ABCA1*; subsequently, 2.8% ApoB-depleted plasma were added to the efflux experiment for 4 hours. Acyl-CoA cholesterol acyltransferase inhibitor (2 µg/mL) was used in all steps. For THP-1 macrophage CEC, THP-1 cells were differentiated into macrophage by treatment with 100 nM phorbol 12-myristate 13-acetate for 24 hours. Subsequently, THP-1 macrophages were loaded with 1 µCi ^3^H-Cholesterol and 50 µg/mL acetylated LDL for 24 hours. After that, 2% ApoB-depleted plasma were used as cholesterol acceptor for 5 hours. In this technique, cholesterol efflux mediated by all pathways were determined. ApoB-depleted plasma was employed as cholesterol acceptor; consequently, in both methods, HDL CEC were assessed. Radioactivity was measured by liquid scintillation counting. CEC was calculated using the following equation: (radioactivity in efflux medium/total radioactivity in cells and medium) × 100. Efflux of experiment without acceptor (ApoB-depleted plasma) was subtracted from the individual efflux.

Furthermore, all efflux assays were carried out in duplicate. Also, a standard ApoB-depleted plasma was used in each plate and individual values normalized to values of the standard ApoB-depleted plasma were obtained; therefore, there was no specific unit for CEC. The inter and intra-assay coefficient of variation (CV) of these methods were 6.1% and 7.2% for cAMP-treated J774 CEC and 6.9% and 7.4% for THP-1 macrophage CEC, respectively.

### Plasma activity levels of LCAT, PLTP and CETP

Plasma activity levels of PLTP and CETP were measured employing activity assay kits (Biovision, USA) according to the manufacturer’s protocols. The reaction mixture contained a donor molecule that was a fluorescent self-quenching neutral lipid as well as an acceptor molecule. Three µL of plasma sample was added to the reaction mixture. PLTP-mediated transfer from donor to acceptor resulted in an increase in fluorescence intensity with an excitation wavelength of 465 nm and emission of 535 nm as read by the fluorescent plate reader. CETP activity was measured by a protocol, the same as PLTP. Plasma LCAT activity level was measured with a commercial kit (Roar Biomedical Inc., USA) according to the manufacturer’s protocol. Four µL of samples were incubated briefly for 5 h at 37 °C with reaction reagent containing a fluorescently labeled substrate. Fluorescently labeled substrate hydrolysis by LCAT resulted in a decrease in emission at 470 nm and an increase in emission at 390 nm. The LCAT activity was expressed as the ratio of two emissions (390 nm/470 nm).

### Plasma levels of ApoA-I, ApoB and preβ1-HDL

ELISA kits (Abcam, USA) were employed for determination of ApoA-I and ApoB plasma levels according to the manufacturer’s protocols. Intra and inter-assay analysis were <6% and <7% for ApoA-I and ApoB, respectively. In addition, preβ1-HDL was measured using ELISA kit (Sekisui Medical Co., Tokyo, Japan), as previously described^40^.

### Statistical analysis

Distribution of quantitative data were tested by Shapiro-Wilk. Normal distributed data were presented as mean ± standard deviation (SD) and compared using student’s t-test. Non-normal distributed data were presented as median and inter quartile range (IQR) and tested employing Mann–Whitney U test. Analysis of covariance (ANCOVA) was carried out to remove the effect of confounders. Pearson correlation analysis was conducted to detect the correlation between continuous variables; before correlation analysis, logarithmic transformation was performed on non-normal distributed data. We conducted multivariate linear regression analysis to identify independent predictors of CECs and cIMT. Additionally, binary logistic regression was carried out to evaluate the risk of atherosclerotic plaque based on cAMP-treated J774 CEC.

### Data availability

The datasets generated during and/or analyzed during the current study are available from the corresponding author on reasonable request.

## Electronic supplementary material

Supplementary tables
