# Supplementary material for: Impaired HDL cholesterol efflux capacity in patients with non-alcoholic fatty liver disease is associated with subclinical atherosclerosis
Source: Sci Rep. 2018 Aug 3;8:11691. doi: 10.1038/s41598-018-29639-5 (PMC6076293; doi:10.1038/s41598-018-29639-5)
Supplement: Supplementary file 1 — Supplementary tables [file 41598_2018_29639_MOESM1_ESM.pdf]

Impaired HDL cholesterol efflux capacity in patients with non-alcoholic fatty liver disease is associated with subclinical atherosclerosis

Reza Fadaei[1], Hossein Poustchi[2,3], Reza Meshkani[1], Nariman Moradi[4,5], Taghi Golmohammadi[1*] and Shahin Merat[2,3]*

1. Department of Clinical Biochemistry, Faculty of Medicine, Tehran University of Medical Sciences, Tehran, Iran

2. Liver and Pancreatobiliary Diseases Research Center, Digestive Diseases Research Institute, Tehran University of Medical Sciences, Tehran, Iran

3. Digestive Disease Research Center, Digestive Disease Research Institute, Tehran University of Medical Sciences, Tehran, Iran

4. Department of Clinical Biochemistry, Faculty of Medicine, Kurdistan University of Medical Sciences, Sanandaj, Iran

5. Department of Clinical Biochemistry, Faculty of Medicine, Iran University of Medical Sciences, Tehran, Iran

Supplementary Table S1. Age adjusted correlation of cAMP-treated J774 CEC and THP-1 macrophage CEC with anthropometric and metabolic profiles.

Variables	cAMP-treated J774 CEC			THP-1 macrophage CEC		
	All Participant	Controls	NAFLD	All Participant	Controls	NAFLD
BMI	-0.295*	0.093	-0.134	-0.264*	-0.245	-0.074
FBG	-0.200	-0.071	-0.279*	-0.035	0.113	-0.110
Insulin	-0.266*	0.187	-0.034	-0.155	0.065	-0.05
HOMA-IR	-0.281*	0.193	-0.073	-0.148	0.097	-0.05
TG	-0.174	-0.151	-0.026	-0.120	-0.158	-0.017
TC	-0.196	-0.247	-0.053	0.027	-0.020	0.145
LDL-C	-0.124	-0.176	0.030	0.071	-0.064	0.237
HDL-C	0.435**	0.394*	0.359**	0.534**	0.564**	0.452**
SBP[a]	-0.148	-0.281	0.098	-0.033	0.200	-0.048
DBP	-0.163	-0.098	-0.043	-0.091	-0.041	-0.013
Urea nitrogen	-0.053	-0.179	0.063	0.018	-0.09	0.062
Creatinine	-0.047	0.059	0.064	0.030	-0.036	0.171
AST[a]	-0.131	0.334	-0.084	-0.075	0.277	-0.067
ALT[a]	-0.173	0.294	-0.069	-0.157	0.213	-0.139
ALP	-0.015	-0.029	0.022	-0.040	0.000	-0.057
ɣ-GT[a]	-0.157	0.227	-0.055	-0.098	0.088	-0.050
LS[a]	-0.324**	0.186	-0.315*	-0.086	-0.173	0.196
ApoB	-0.099	0.151	0.056	-0.086	-0.156	0.119
ApoA-I	0.667**	0.529**	0.536**	0.316**	0.234	0.127
preβ1-HDL	0.486**	0.266	0.460**	0.409**	0.251	0.383**
CETP activity	-0.137	0.05	0.063	-0.051	0.029	0.058
PLTP activity	0.040	0.029	0.076	-0.026	-0.010	-0.029
LCAT activity	0.339**	0.137	0.389**	0.330**	0.276	0.305*
cAMP-treated J774 CEC	-	-	-	0.405*	0.296	0.30*

*p<0.05 and **p<0.01.
a: Logarithmic transformation was performed.

Supplementary Table S2. Age adjusted correlation of cIMT with anthropometric and metabolic profiles.

Variable	All Participant	Controls	NAFLD
BMI	0.207	0.312	0.017
FBG	0.056	-0.126	0.135
Insulin	0.248*	-0.179	0.223
HOMA-IR	0.260*	-0.203	0.249
TG	0.237*	0.017	0.237
TC	0.249*	0.051	0.263
LDL-C	0.185	0.059	0.193
HDL-C	-0.334**	-0.369*	-0.261
SBPa	0.088	0.433*	-0.117
DBP	0.086	0.234	-0.063
Urea nitrogen	0.067	0.324	-0.044
Creatinine	0.061	-0.187	0.077
ASTa	0.176	-0.019	0.123
ALTa	0.108	-0.101	0.023
ALP	-0.045	0.275	-0.173
γ-GTa	0.06	-0.058	-0.138
LSa	0.233*	-0.049	0.176
ApoB	0.179	0.087	0.111
ApoA-I	-0.457**	-0.404*	-0.409**
preβ1-HDL	-0.389**	-0.200	-0.362**
CETP	0.096	-0.053	0.067
PLTP	0.172	0.170	0.178
LCAT	-0.253*	-0.394*	-0.162
cAMP-treated J774 CEC	-0.525**	-0.445*	-0.523**
THP-1 macrophage CEC	-0.269*	-0.211	-0.203

*p<0.05 and **p<0.01.
a: Logarithmic transformation was performed.
